# Is Audio Computer-Assisted Self-Interview (ACASI) Useful in Risk Behaviour Assessment of Female and Male Sex Workers, Mombasa, Kenya?

**DOI:** 10.1371/journal.pone.0005340

**Published:** 2009-05-01

**Authors:** Elisabeth M. van der Elst, Haile Selassie Okuku, Phellister Nakamya, Allan Muhaari, Alun Davies, R. Scott McClelland, Matthew A. Price, Adrian D. Smith, Susan M. Graham, Eduard J. Sanders

**Affiliations:** 1 Centre for Geographic Medicine Research-Coast, Kenya Medical Research Institute (KEMRI), Kilifi, Kenya; 2 Departments of Medicine and Epidemiology, University of Washington, Seattle, Washington, United States of America; 3 International AIDS Vaccine Initiative, New York, New York, United States of America; 4 Department of Public Health & Primary Care, University of Oxford, Headington, United Kingdom; 5 Departments of Medicine, University of Washington, Seattle, Washington, United States of America; 6 Centre for Clinical Vaccinology and Tropical Medicine, University of Oxford, Headington, United Kingdom; McGill University Health Center, Montreal Chest Institute, Canada

## Abstract

**Background:**

Audio computer-assisted self-interview (ACASI) may elicit more frequent reporting of socially sensitive behaviours than face-to-face (FtF)-interview. However, no study compared responses to both methods in female and male sex workers (FSW; MSW) in Africa.

**Methodology/Principal Findings:**

We sequentially enrolled adults recruited for an HIV-1 intervention trial into a comparative study of ACASI and FtF-interview, in a clinic near Mombasa, Kenya. Feasibility and acceptability of ACASI, and a comparative analysis of enrolment responses between ACASI and FtF on an identical risk assessment questionnaire were evaluated. In total, 139 women and 259 men, 81% of eligible cohort participants, completed both interviews. ACASI captured a higher median number of regular (2 vs. 1, p<0.001, both genders) and casual partners in the last week (3 vs. 2, p = 0.04 in women; 2 vs. 1, p<0.001 in men). Group sex (21.6 vs. 13.5%, p<0.001, in men), intravenous drug use (IDU; 10.8 vs. 2.3%, p<0.001 in men; 4.4 vs. 0%, p = 0.03 in women), and rape (8.9 vs. 3.9%, p = 0.002, in men) were reported more frequently in ACASI. A surprisingly high number of women reported in ACASI that they had paid for sex (49.3 vs. 5.8%, p<0.001). Behaviours for recruitment (i.e. anal sex, sex work, sex between males) were reported less frequently in ACASI. The majority of women (79.2%) and men (69.7%) felt that answers given in ACASI were more honest. Volunteers who were not able to take ACASI (84 men, and 37 women) mostly lacked reading skills.

**Conclusions/Significance:**

About 1 in 5 cohort participants was not able to complete ACASI, mostly for lack of reading skills. Participants who completed ACASI were more likely to report IDU, rape, group sex, and payment for sex by women than when asked in FtF interview. ACASI appears to be a useful tool for high risk behaviour assessments in the African context.

## Introduction

Audio computer-assisted self-interview (ACASI), which is known to capture some sensitive behaviours more reliably in high risk populations in developed nations [1], has not been evaluated in populations recruited for HIV-1 prevention trials in Africa. As such populations are purposefully selected for presumed ‘high-risk’ behaviour, interviewer attitudes may impact socially desirable responses of the interviewee, especially because socially stigmatised behaviours such as male same sex behaviour, or anal sex practice, are rarely assessed in Africa [2], [3].

Research on computerized interviewing in the USA has shown that replacing the interviewer with a computer can provide conditions, including privacy and the perception of anonymity, that facilitate reliable and frank reporting, thereby increasing reports of sensitive behaviour in surveys of the general population [4], [5], adolescents [1], [6], and injection drug users (IDU) [7], [8]. In addition to reducing social desirability bias, ACASI has several advantages over face-to-face (FtF)-interview: it standardises data collection by using recorded audio question tracks and captures data directly into a database, allowing for automated skip patterns and built in logic checks. The use of ACASI has been recommended for socio-behavioural research in developing countries [1], [9].

Perhaps due to its cost and technological complexity, the use of ACASI in African research studies has been low. Comparison studies of ACASI and FtF-interview in the general population in Zimbabwe and in 4 other developing countries suggested that participants are at ease with completing ACASI, and preferred a computer for answering sensitive questions, although low literacy may pose problems [10], [11]. Among adolescents in Kenya, substantial differences between interview methods were reported for questions related to premarital sex, although not always in the expected direction [12], [13]. Ongoing studies targeting adolescents in rural Malawi aim to evaluate which method of interview elicits increased reports of sexual risk behaviour, including an assessment of the impact of order of the interview mode on responses [14].

To our knowledge, no study has compared responses by ACASI with FtF-interview in high risk populations recruited for HIV-1 prevention trials in Africa. In Mombasa, Kenya, male and female sex workers (MSW, FSW) have been invited to join an ongoing cohort study to monitor HIV-1 seroincidence in preparation for studies of new HIV-1 prevention interventions [15], [16]. Previously, at enrolment to the cohort study, HIV risk behaviours have been established through FtF-interview. For this study, we invited cohort participants to also take ACASI at their enrolment visit, and compared risk behaviour in volunteers who had undergone both ACASI and FtF-interview. Our study was exploratory and aimed at hypothesis generation and assessment of contextual acceptability and feasibility of ACASI in FSW and a large group of MSW who have sex with men. Both sex work and homosexual sex are illegal in Kenya. We assumed ACASI to be helpful in detecting behaviours that may be under-reported in FtF-interview due to social desirability bias. We also assumed that the order of the interview mode might influence responses given in either method. Hence we crossed over the order of interview methods. All interviews were taken on the same day and prior to HIV-1 testing.

## Results

### Eligibility and feasibility of ACASI

Eligibility was assessed in 519 newly recruited cohort participants (343 men and 176 women). Of these, 121 (23%) volunteers (84 men, 37 women, p = 0.3) were not able to take ACASI; 72 (59%) were unable to read, 25 (21%) experienced a technical computer problem while taking ACASI, 16 (13%) were not willing, and 8 (7%) were initially willing but declined after waiting too long. Excluding technical failures, 398 (81% of 494) consecutive cohort participants were able and willing to complete ACASI.

Socio-demographic characteristics and laboratory confirmed STI for participants and non-participants are shown in table 1. HIV-1 prevalence did not differ between participating and non-participating men and women. Not surprisingly, both non-participating genders had a similar low education attainment. Non-participating men presented more often with non-specific urethritis at enrolment, and a larger proportion was unemployed. There were no differences in key risk behaviour comparisons established in FtF interview (i.e. gender of sex partner, anal sex, been paid, or having paid for sex, partner counts, group sex, rape, and IDU) between participating and non-participating men. Non-participating women presented more often with syphilis at enrolment, and a larger proportion had no prior knowledge of their HIV-1 status. Non-participating women more frequently reported anal sex in the past 3 months (62.2 vs. 16.8%, p = 0.05), and recalled a higher number of recent casual partners (median 4 vs. 2, p = 0.003).

**Table 1:** Characteristics of Audio Computer-Assisted Self-interview (ACASI)-participating and non-participating study participants, at cohort enrolment, Kenya, 2008.

Socio demographic characteristics & Laboratory confirmed infections, at screening	Participating men N = 259	Non-participating men N = 84	P - value
	% (n) or median	% (n) or median	
Age, yrs			
Median	27 (23–32)	27 (22–34)	0.9
Level of education			
Median years in education (IQR)	9 (8–12)	4 (0–8)	<0.001
Marital status			
Single	80.3 (208)	82.1 (69)	
Current marriage	9.7 (25)	5.9 (5)	
Separated/widowed	10.0 (26)	11.9 (10)	0.5
Employment			
None	43.4 (112)	66.7 (56)	
Self	34.8 (90)	22.6 (19)	
Formal	21.7 (56)	10.7 (9)	0.001
Knowledge of HIV status			
Don't know	73.3 (190)	69.1 (58)	
Know positive	1.9 (5)	2.4 (2)	
Know negative	24.7 (64)	28.6(24)	0.7
HIV-1 status			
Negative	80.3 (208)	80.9 (68)	
Positive	19.7 (51)	19.1 (16)	0.9
Syphilis status			
Negative	96.9 (251)	97.6 (820)	
Positive	3.1 (8)	2.4 (2)	0.7
Non-specific urethritis, (n = 95)	4.7 (3/64)	19.4 (6/31)	0.02
Non-specific proctitis, (n = 89)	8.3 (6/72)	0 (0/17)	0.2

Socio demographic characteristics & Laboratory confirmed infections, at screening	Participating women N = 139	Non-participating women N = 37	
	% (n) or median	% (n) or median	
Age, yrs			
Median	28 (24–32)	28 (24–35)	0.8
Level of education			
Median years in education (IQR)	9 (8–12)	4 (0–7)	<0.001
Marital status			
Single	68.4 (95)	51.4 (19)	
Current marriage	5.0 (7)	5.4 (2)	
Separated/widowed	26.6 (37)	43.2 (16)	0.1
Employment			
None	39.6 (55)	45.9 (17)	
Self	56.8 (79)	54.1 (20)	
Formal	3.6 (5)	0	0.4
Knowledge of HIV status			
Don't know	33.0 (46)	54.1 (20)	
Know positive	8.6 (12)	10.8 (4)	
Know negative	58.3 (81)	35.1 (13)	0.04
HIV-1 status			
Negative	70.5 (98)	62.2 (23)	
Positive	29.5 (41)	37.8 (14)	0.3
Syphilis status			
Negative	97.8 (136)	86.5 (32)	
Positive	2.2 (3)	13.5 (5)	0.003
Non-specific cervicitis, (n = 143)	18.6 (21/113)	26.7 (8/30)	0.3
Non-specific proctitis, (n = 19)	8.3 (1/12)	0 (0/7)	0.4

A total of 259 men and 139 women completed both interview methods, and were similar in age, education attainment, and unemployment status, although more men had formal employment (table 1). The majority (73.0%) of men did not know their HIV-1 status while 66.9% of women had taken an HIV test before.

### Acceptability of ACASI

An almost equal proportion, 33.3% in women, and 30.8% in men, had used a computer previously (table 2). Over 90% in both groups found the questions easy to understand and were comfortable in answering. Overall, almost 80% of women and 70% of men felt that answering questions in ACASI would give more honest answers, 11.7% of women, and 16.0% of men felt the methods were about the same, and 9.2% of women, and 14.3% of men felt answers in ACASI would be less honest than in a FtF-interview, p = 0.16. These proportions were similar regardless of the order of methods, with the exception that no woman who took ACASI after FtF believed that ACASI would provide less honest answers (data not shown). Mean duration to complete ACASI was almost double the duration of the FtF-interview for both women and men (31.2 vs. 15.9 minutes for men; 35.7 vs. 18.9 minutes for women, p = 0.001). Women took longer than men to complete both FtF- and ACASI- interviews, by 3 and 5 minutes, respectively. Men and women experienced the duration of ACASI as just right (67.1 vs. 62.3%) or too short (11.9 vs. 17.5%), while 20% of both groups felt that ACASI took too long.

**Table 2:** Acceptability of Audio Computer-Assisted Self-Interview (ACASI) of 139 women and 259 men, at cohort enrolment, Kenya, 2008.

Acceptability of ACASI	Women N = 139	Men N = 259
	% (n) or median	% (n) or median
Computer use prior to ACASI		
No	66.7 (78)	69.2 (166)
Yes	33.3 (39)	30.8 (74)
Understanding of questions		
Easy	94.2 (113)	93.8 (226)
Difficult	5.8 (7)	6.2 (15)
Self perception while answering questions		
Comfortable	95.8 (114)	92.2 (225)
Not so comfortable	3.4 (4)	6.6 (16)
Not comfortable at all	0.8 (1)	1.2 (3)
Honesty of ACASI vs. FtF		
More honest	79.2 (95)	69.7 (170)
About the same	11.7 (14)	16.0 (39)
Less honest	9.2 (11)	14.3 (35)
Duration of ACASI^2^		
Just right	67.1 (90)	62.3 (160)
Too short	11.9 (16)	17.5 (54)
Too long	20.9 (28)	20.2 (52)
Duration ACASI (min)	35.7	31.2
Duration FtF-interview (min)	18.9	15.9

2 Missing values in acceptability questions during start up period (March–April 2006; 22 women and 19 men).

### Comparisons of sexual risk behaviour, substance misuse, and violence

**Figure 1:**
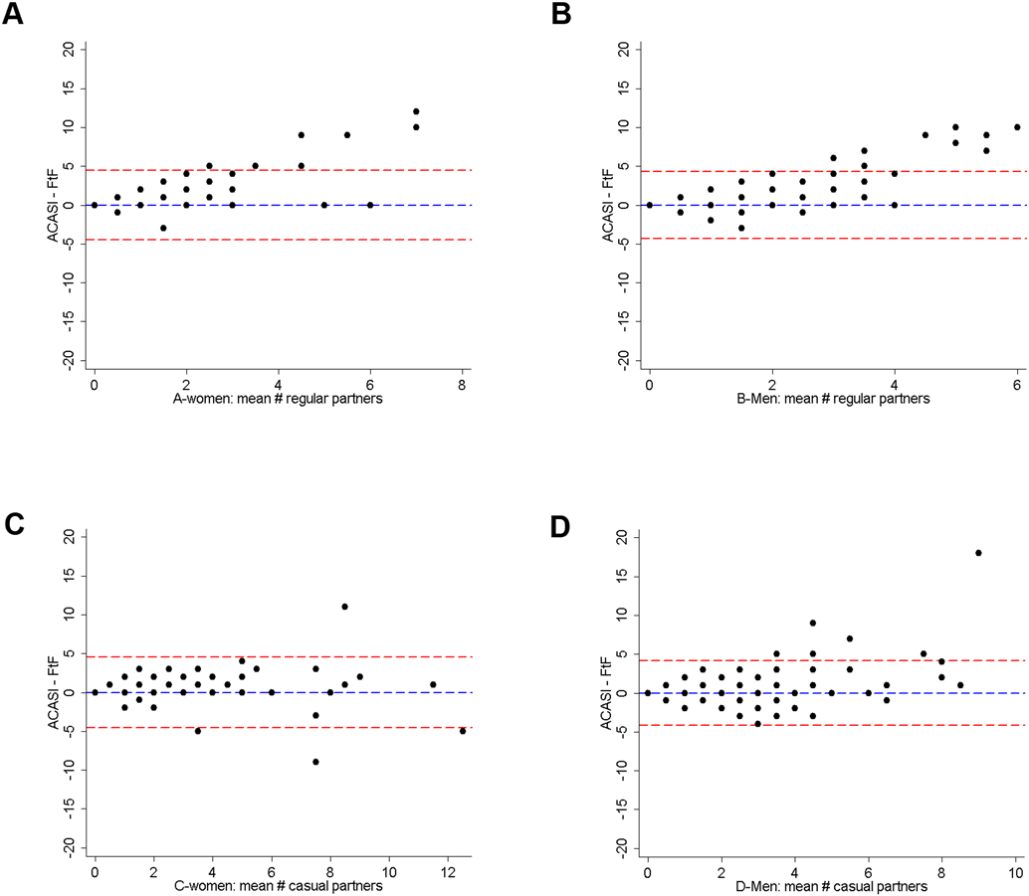
A–D: Bland-Altman plots. The plots show differences between number of regular and casual partners in the last week collected by Audio Computer-Assisted Self-Interview (ACASI) and Face-to-Face interview (FtF) in the same volunteer on the same day, Mombasa, Kenya. The Y-axis presents the difference in partner counts in ACASI and FtF; the X-axis presents the mean number of partners in both methods. Top row: regular partners in the last week in women (A) and men (B); bottom row: casual partners in the last week in women (C) and men (D). Horizontal lines: mean of difference with the 95% limits of agreement. Interpretation figures A and B: ACASI captures a higher number of regular partners in women and men when the average number of regular partners captured in both interview methods is about two or higher. Interpretation figures C and D: For women, the variability between methods is more constant. For men, there is more variation between methods.

ACASI captured a significantly higher median number of casual sex partners in women (3 vs. 2, table 3) and men (2 vs. 1, table 4), with excellent agreement between both interview methods in women, and good agreement in men. Agreement between methods for reported regular sex partners for women and men was poor. Bland Altman plots (figure 1) show the average of the reported number of partners (regular or casual) by both methods versus the difference between reported number of partners by ACASI minus FtF-interview for women and men. For both men and women, ACASI was more sensitive in capturing a higher regular partner number (2 or more in the last week), while FtF-interview captured a slightly higher report of regular partner numbers when lower numbers (<2) were reported. Bland-Altman plots demonstrate that for each increase in average partner number participants reported more regular partners by ACASI (figure 1, A and B). For casual partners, there appears to be no such increase between methods (figure 1, C and D).

**Table 3:** Comparison of characteristics in 139 women in Audio Computer-Assisted Self-Interview (ACASI) and Face-to-Face (FtF) interview, at cohort enrolment, Kenya, 2008.

Sexual risk behaviour characteristics, and substance use, in women	ACASI N = 139	FtF N = 139	P	Agreement^1^ (95% CI)
	% (n) or Median (IQR)	% (n) or median (IQR)		
Sex partners in past week^2^				
Regular partner(median, IQR)	2 (1–3)	1 (0–1)	**<0.001**	**0.22 (0.04–0.38)**
Casual partner(median, IQR)	3 (1–5)	2 (1–4)	**0.04**	**0.78 (0.69–0.85)**
New partner (median, IQR)	2 (1–4)	1 (0–3)	**<0.001**	**0.68 (0.57–0.77)**
Sex partners in past month^3^				
Regular partner(median, IQR)	2 (1–5)	1 (0–1)	0.21	0.30 (0.16–0.44)
Casual partner(median, IQR)	2 (1–7)	1(0–4)	1.0	0.78 (0.51–0.91)
New partner (median, IQR)	2 (0–4)	1(0–2)	1.0	0.36 (−0.15–0.72)
Condom use, always				
with regular partner, past week	46.0 (64)	48.2 (67)	0.75	0.44 (0.29–0.58)
with casual partner, past week	55.6 (55)	57.6 (57)	0.23	0.40 (0.24–0.56)
with anal sex, previous 3 months	18.5 (10)	24.6 (15)	0.18	0.58 (0.56–0.66)
Partner type^4^				
Men only	90.8 (99)	92.7 (101)	0.69	
Men and women	8.3 (9)	7.3 (8)	1.0	
Women only	0.9 (1)	-	1.0	0.69 (0.58–0.71)
Anal sex, in past 3 months				
No	60.4 (84)	56.1 (78)		
Yes	39.6 (55)	43.9 (61)	0.15	0.82 (0.72–0.92)
Received cash for sex, in past 3 months				
No	12.9 (18)	4.3(6)		
Yes	87.1(121)	95.7 (133)	**0.01**	**0.11 (−0.09–0.31)**
Paid for sex, in past 3 months^5^				
No	50.7 (70)	94.2 (131)		
Yes	49.3 (68)	5.8 (8)	**<0.001**	**0.03 (−0.05–0.11)**
Group sex, in past 3 months				
No	77.0 (107)	81.3 (113)		
Yes	23.0 (32)	18.7 (26)	0.26	0.57 (0.39–0.73)
Substance misuse, in last month, daily				
Cigarettes	19.6 (27)	20.3 (28)	1.0	0.81 (0.71–0.85)
Marijuana	12.3 (17)	13.0 (18)	1.0	0.83 (0.80–0.87)
Any alcoholic drink	26.8 (37)	21.7 (30)	0.1	0.70 (0.57–0.83)
Drunk (daily)	25.2 (27)	24.3 (26)	1.0	0.37 (0.29–0.40)
Used IV drugs in past 3 months^5^				
No	95.7 (132)	100 (139)		
Yes	**4.4 (6)**	**0(0)**	**0.03**	**0.00 (− −1.0)**
Violence, been raped in past 3 months^5^				
No	93.5 (129)	95.4 (132)		
Yes	6.5(9)	4.4 (6)	0.4	0.65 (0.36–0.93)

IQR inter quartile range.

1 Spearman or kappa statistic.

2 Included those active in past week: 126 women in ACASI; 125 women in FtF.

3 Included those not active in past week: 13 women in ACASI; 14 in FtF.

4 Variable missing for 30 women who completed the study before this question added.

5 Missing values in 1 woman.

**Table 4:** Comparison of characteristics in 259 men in Audio Computer-Assisted Self-Interview (ACASI) and Face-to-Face (FtF) interview, at cohort enrolment, Kenya, 2008.

Sexual behaviour Characteristics, and substance use, in men	ACASI N = 259	FtF N = 259	P	Agreement 1 (95% CI)
	% (n) or Median IQR	% (n) or median IQR		
Sex in past week^2^				
Regular partner(median, IQR)	2 (1–3)	1 (0–1)	**<0.001**	**0.23 (0.08–0.36)**
Casual partner(median, IQR)	2 (1–3)	1 (0–3)	**<0.001**	**0.61 (0.51–0.69)**
New partner (median, IQR)	1 (1–3)	1 (0–2)	**<0.001**	**0.56 (0.45–0.65)**
Sex in past month^3^				
Regular partner(median, IQR)	2 (1–3)	1 (0–1)	**0.02**	**0.31 (−0.55–0.02)**
Casual partner(median, IQR)	3 (1–5)	1 (0–3)	0.55	0.41 ((0.14–0.62)
New partner (median, IQR)	1 (0–3)	0(0–2)	1.0	0.52 (0.28–0.70)
Condom use, always				
with regular partner, past week	37.7 (46)	23.8 (29)	**0.006**	**0.34 (0.17–0.51)**
with casual partner, past week	41.3 (57)	35.5 (49)	1	0.49 (0.39–0.60)
with anal sex (previous 3 months)	15.4 (28)	19.3 (43)	0.1	0.67 (0.62–0.74)
Partner type^4^				
Men only	30.1 (71)	32.2 (76)	0.49	
Men and women	61.0 (144)	66.7 (155)	0.14	
Women only	8.9 (21)	2.1 (5)	<0.001	0.60 (0.59–0.69)
Anal sex, in past 3 months				
No	22.0 (57)	7.3 (19)		
Yes	**78.0 (202)**	**92.7 (240)**	**<0.001**	**0.41 (0.27–0.55)**
Participation in anal sex				
Any insertive	**63.3 (164)**	**72.6 (188)**	**<0.001**	**0.56 (0.48–0.68)**
Any receptive	**44.8 (116)**	**57.5 (149)**	**<0.001**	**0.64 (0.55–0.73)**
Both insertive and receptive	**29.7 (77)**	**37.5 (97)**	**0.006**	**0.59 (0.49–0.69)**
Received cash for sex, in past 3 months				
No	35.5 (92)	28.6 (74)		
Yes	64.5 (167)	71.4 (185)	**0.006**	**0.65 (0.55–0.75)**
Paid for sex, in past 3 months				
No	54.8 (142)	60.6 (157)		
Yes	45.2(117)	39.4 (102)	0.09	0.46 (0.35–0.57)
Group sex, in past 3 months				
No	78.4 (203)	86.5 (224)		
Yes	**21.6 (56)**	**13.5 (35)**	**<0.001**	0.54 (0.41–0.67)
Substance misuse, in last month, daily use				
Cigarettes	50.2 (130)	50.2 (130)	1.0	0.75 (0.73–0.78)
Marijuana	23.2 (60)	22.4 (58)	0.8	0.74 (0.69–0.81)
Any alcoholic drink	12.4 (32)	11.6 (30)	0.8	0.67 (0.53–0.81)
Drunk (daily)	16.8 (26)	15.5 (24)	0.8	0.39 (0.33–0.46)
Used IV drugs in past 3 months				
No	89.2 (231)	97.7 (253)		
Yes	**10.8 (28)**	**2.3 (6)**	**<0.001**	0.33 (0.13–0.53)
Violence, been raped in past 3 months				
No	91.1 (236)	96.1 (249)		
Yes	**8.9 (23)**	**3.9 (10)**	**0.002**	0.46 (0.24–0.67)

1 Spearman correlation coefficient or kappa statistic.

2 Included those active in past week: 198 men in ACASI; 206 in FtF.

3 Included those not active in past week: 61 men in ACASI; 53 in FtF.

4 Variable not asked in 23 subjects in ACASI.

Reported consistent (i.e. 100%) condom use with casual sex partners in the past week was similar for both methods (women: 55.6 ACASI vs. 57.6% FtF, men: 41.3 ACASI vs. 35.5% FtF). In ACASI, reported consistent condom use during anal sex over the last 3 months, was substantially lower for both women (18.5 ACASI vs. 24.6% FtF) and men (15.4 ACASI vs. 19.3% FtF, tables 3 and 4).

Reported partner gender preference for women and men was similar in ACASI and FtF-interview. Fewer women reported anal sex practice (39.6 vs. 43.9, p = 0.15), and fewer women reported having received cash for sex (87.1 vs. 95.7%, p = 0.01) in ACASI than in FtF-interviews; these behaviours were recruitment criteria for cohort enrolment. Almost half of the women reported having paid for sex themselves in ACASI, while few admitted to this in FtF-interview (49.3 ACASI vs. 5.6% FtF, p<0.001).

Behaviours that were recruitment criteria for cohort enrolment, including anal sex practice, anal receptive role-taking, and having received cash for sex in the last 3 months, were reported significantly less frequently in ACASI than in FtF interviews with men. However, more men in ACASI than in FtF-interview reported participating in group sex (21.6 vs. 13.5%, p<0.001) and having paid for sex themselves (45.2 vs. 39.5%, p = 0.09).

ACASI and FtF-interview had excellent agreement for daily cigarette and marijuana use in women and men, good agreement for daily alcohol use, but poor agreement for being drunk daily. A lower proportion of women and men said in ACASI that they always used alcohol before sex (14.0% vs. 31.8%, p<0.001, for women; 14.2 vs. 40.0%, p<0.001, for men, data not shown).

IV drug use in the last three months was reported by six women in ACASI, but not at all in FtF-interview (4.4 vs. 0%, p = 0.03). Similarly, a higher proportion of men reported IV drug use in ACASI than FtF (10.8 vs. 2.3%, p<0.001). Both men and women reported having been raped (in the last three months) more often in ACASI compared to FtF (women: 6.6 vs. 4.4%, p = 0.4, men 8.9 v 3.9%, p = 0.002).

### Order of interview mode

The order of interview mode for categorical variables was assessed for three risk behaviours that were significantly different between methods in women (i.e. received cash for sex, paid for sex, and IV drug use, table 3) and eight in men (i.e. sex partner gender, anal sex and role taking, received cash for sex, paid for sex, group sex, IV drug use and rape, table 4).

Of 95 women who took ACASI first, 16 (16.8%) denied receiving cash for sex in ACASI, but 15 of these 16 women subsequently reported this activity in FtF-interview. Of the 44 women who took ACASI after FtF-interview, only 2 (4.5%) denied transactional sex in ACASI, while one of the two women had reported this during the preceding FtF-interview (16.8 vs. 4.5%, p = 0.04). An equal proportion of women admitted to having paid another person for sex independent of when ACASI was taken (48.8 vs. 49.5%, p = 0.9). No differences were found for IV drug use by order of interview mode in women (data not shown).

In men, the order of the interview method mattered only for sex partner gender in men who had self-identified as men who have sex with men (MSM) in the FtF-interview (91.5% of all men). Of 140 MSM (by FtF-standard) who took ACASI before the FtF-interview, 14 (10.0%) admitted to have sex with women only to the computer, while none said so in the FtF-interview that they did after ACASI. Of 91 MSM who took ACASI after the FtF-interview (all reported to be either homosexual, or bisexual to the counsellor), 2 (2.2%) changed their sex partner preference in ACASI, and reported sex with women only (10.0 vs.2.2%, p = 0.02).

## Discussion

This is the first study comparing ACASI with FtF-interview in African high risk populations. We compared responses to an identical risk assessment questionnaire in 81% of the consecutively enrolled cohort participants (139 women, 259 men). Almost one out of five newly enrolled cohort participants (84 men and 37 women) were not able to take ACASI, mostly for lack of reading skills. Risk behaviour assessed in FtF-interview between non-ACASI and ACASI participants was not different in men; but women unable to use ACASI more frequently reported anal sex in the previous 3 months, and a higher number of recent casual partners.

The majority of women and men felt that answers given in ACASI were more honest. Over 90% of women and men were comfortable with ACASI, and, -although ACASI was on average twice as long as FtF-interview-, the majority of the volunteers had no objection to the longer interview time. Noteworthy is a recent assessment in ACASI of intentional provision of misinformation in FtF-interview by female microbicide trial participants in South Africa. Almost 80% had done this at least once, for reasons including politeness, to avoid criticism or seek praise, and embarrassment [17].

We explored responses to an identical questionnaire offered in ACASI and in FtF-interview and assessed the impact of the order of the interview mode. Among participants who took both interview methods, ACASI and FtF-interview had excellent agreement for less sensitive behaviours in both men and women, as demonstrated by reports on daily cigarette or marijuana use. Group sex, IDU, (in men), and rape (in men) were significantly more frequently reported in ACASI. A similar trend towards higher responses in ACASI for group sex and rape was seen in women. Thus, ACASI led to more frequent reporting of sensitive behaviours that were not linked to recruitment criteria.

In contrast, potentially stigmatising behaviours that were also recruitment criteria were reported less frequently in ACASI compared to FtF-interviews. Transactional sex in the 3 months prior to cohort enrolment in men and women were less likely to be reported in ACASI. Moreover, 38 (14.7%) of men who reported anal sex in FtF- interviews did not report anal sex in ACASI, and MSM who took ACASI before FtF-interview were more likely to deny same sex behaviour than men who took ACASI after FtF-interview.

As African research sites are often located in settings under-served by formal health care [18], and prospective trial populations may misrepresent or over-report behaviour to ensure access to health care benefits through participation in research programmes, it seems probable that there is some misclassification at cohort enrolment for both MSM and transactional sex work in our cohort. Our study evaluating the effect of the order of interview mode on highly stigmatised and sensitive behaviours among 398 high-risk volunteers (most of whom were sex workers), suggested that ACASI is better conducted prior to FtF-assessment. Failure to disclose risk, or the reverse (i.e.overstating risk, as we suspect was the case in our study) would have significant implications for participant selection and behaviour tracking during intervention trials [17], [19].

That almost half of the women admitted to having paid for sex in ACASI was a surprise, and initially doubted. A focus group discussion conducted in 2008 with 13 women who admitted to payment for sex in ACASI suggested that this behaviour was indeed common, and gave some women a sense of control over the sex they purchased. Furthermore, a prospective sexual behaviour study conducted among 81 male sex workers recruited from the same cohort population, revealed that 68% of 238 female partners had paid for sex [20]. The finding that FSW purchase sex themselves has not been previously reported to our knowledge.

The crux to establishing high risk cohorts for HIV-1 prevention studies is a reliable and accurate assessment of volunteers' risk behaviour, that is sensitive to marginalised and stigmatised behaviours, and is conducive to truthful reporting. Surprisingly, ACASI has not been thoroughly evaluated among sex workers. The few ACASI comparison studies in Africa involved adolescents or general population, and firm conclusions of its usage have not been drawn, either because sample sizes were too small, or study findings were contradicting expectations [10], [11], [13]. A randomised crossover study of ACASI and computer-assisted personal interview (CAPI) in 445 adult volunteers from China, India, Peru, Russia, and Zimbabwe, found few differences in responses between methods, except for China, where volunteers gave a significantly higher response to some sensitive questions (i.e. ever had sex, number of partners, and unprotected acts) in CAPI [11]. Similar to our study, ACASI took twice as long on average in 4 of the 5 countries, and volunteers preferred a computer to an interviewer for answering sensitive questions [11]. ACASI's longer duration to complete suggest that volunteers take more time to consider their responses, which makes more honest reporting probable. ACASI enabled women, more than men, to report a higher median number of regular and casual sex partners.

Why then is ACASI not more widely used in research settings in Africa? While start-up costs or lack of systems capacity may pose some challenges, trial sites are often able to access financial and technical support from internal and external sources. That ACASI is merely used experimentally and is not more established in African settings is partly due to unfamiliarity with the interview method, but more likely based on mixed and sometimes contradicting findings. Indeed, in a recent prospective, randomised, cross-over design of 655 women enrolled in a study on hormonal contraceptive use with ACASI and FtF-interview in Zimbabwe, ACASI yielded higher reports on several reproductive health behaviours but discrepancies between self-reports and clinical data highlighted persistent measurement challenges [21]. Minnes et al. suggest that epidemiological studies should use multiple data sources, where possible, to estimate the range and direction of potential bias, and minimize misclassification [22].

We agree that multiple data sources should be used, and prefer ACASI as a screening tool to help identify high risk populations as it elicits sensitive behaviours (ie. IDU and rape) that would otherwise go unreported. We also believe that ACASI provides an environment more conducive to truthful reporting, that may help prospective volunteers avoid overstating their risk (e.g. anal sex, transactional sex).

ACASI has a number of other benefits over FtF-interview, including consistency and standardisation of data collection, and elimination of the need for further data transcription. Admittedly, comparing ACASI-, or FtF-interview, to sexual diary studies kept over a corresponding recall period would have provided a better opportunity to assess accuracy and reliability [22]. Our study also was not designed to test for noninferiority and superiority of ACASI and did not use biomarkers as some others have done. [23], but analysis is ongoing of data collected for a 3-way comparison between ACASI, FtF-interview, and sex partner diaries prospectively kept by 59 MSM cohort participants. A formal evaluation of ACASI as a screening tool to determine eligibility in settings as ours is justified, as planned intervention studies will include Pre-Exposure Prophylaxis that may be especially appealing to prospective volunteers to overstate their sexual risk behaviour and ensure enrolment.

This study has a number of additional limitations. First, our study was not able to distinguish honesty from accuracy; peer mobilization prior to cohort enrolment may have encouraged prospective volunteers to over-report ‘high risk’ behaviour in FTF-interviews. Second, the questionnaire had intrinsic weaknesses such as the assumption that regular and casual partners are clearly distinguishable. It also seems reasonable to assume that some questions were explained by counsellors differently during FtF-interview than in ACASI, and that participant had the opportunity to clarify uncertainties, but we have no documentation of this. Third, the FtF- interviews were performed by a number of different counsellors, and we were not able to gender-match counsellors to clients. It may be that some volunteers were less comfortable with counsellors of the opposite gender or with the interview style of a specific counsellor. Fourth, the study excluded volunteers who were not able to read and may have had higher sexual risk, given that more sexually transmitted infections were diagnosed in these volunteers. A more user friendly version of ACASI could have facilitated use irrespective of education level [24]. Lastly, as in other ACASI comparison studies [14], some discrepant responses (for example, a case of proctitis in a woman who denied anal sex in ACASI, and would on that basis not have been examined) were not fully understood.

Despite these limitations, we feel that ACASI could have an important role in risk behaviour assessments among high-risk populations in Africa. The majority of women (79.2%) and men (69.7%) felt that answers given in ACASI were more honest. Over 90% of women and men were comfortable with ACASI, and, -although ACASI was on average twice as long as FtF-interview-, the majority (80%) of the volunteers had no objection to the longer interview time. ACASI revealed some misclassification of same-sex behaviour, and an over-report of anal sex at cohort enrolment. Largely, ACASI elicited reports of risk behaviours in the same range as FtF-interview, and helped us to capture behaviours hitherto not recognised in our cohort populations. These behaviours were IDU in men and women, rape and group sex in men, and payment for sex by women. Such risk behaviour needs specific risk reduction counselling and further investigation.

## Materials and Methods

### Recruitment of study population

Since July 2005, populations in and around Mombasa at higher risk for HIV infection, including men and women who admitted to transactional sex in the past 3 months, have been targeted for an HIV-1 prevention cohort. Identification and recruitment of prospective study participants has been described elsewhere [16]. In short, peer educators identified volunteers and accompanied them to a Drop-in Centre adjacent to the research clinic. Prospective cohort participants were shown a 25-minute video describing cohort procedures, including a short demonstration of ACASI. Cohort eligibility was assessed by a pre-enrolment counsellor who discussed sexual and other risk behaviour. Cohort inclusion criteria were verified by an enrolment counsellor and were either a self-report of transactional sex, anal sex in the past three months, or same sex behaviour for men. Upon cohort enrolment, participants committed to an HIV-1 test, a risk assessment by standardized FtF-interview, and a medical examination with screening for sexually transmitted infections (STI). Details on cohort procedures, including case definitions used for HIV-1 and STI diagnosis, are described elsewhere [16], [25]. Participants who tested HIV-1 positive at screening were offered follow up into a parallel HIV-1 positive cohort [16].

All study participants provided written informed consent. This study received approval from the National Ethical Review Board.

### Eligibility for ACASI and allocation to interview mode

Newly enrolled cohort participants in the period March 2006–May 2008, who had basic reading skills were invited to undergo both interview methods (ACASI and FtF-interview) on the same day. For ease of clinic organization, participants were either allocated to take ACASI first followed by FtF-interview, or vice versa, in periods of approximately 6 months during the study period. All ACASI and FtF-interviews were conducted before voluntary testing and counselling for HIV-1.

### Risk assessment

We developed an ACASI questionnaire with identical questions to the structured questionnaire used for the FtF- interview. Ordered questions appeared in text on the computer screen, accompanied by a spoken recording in either English or Swahili. Automatic text and voice prompts included standard definitions of terms (e.g. “ a new sex partner is someone you have only known for one week or less”). The questionnaire included questions on number of regular, casual, and new partners in the last week and month, anal sex (practice, role and condom use), gender of their sexual partners (from Sept 2006), transactional sex, substance misuse, and sexual violence.

ACASI study participants were accompanied to a small computer room and instructed in the working of ACASI using socio-demographic questions. Upon completion of these, ACASI was operated alone and in privacy to answer risk assessment questions in either English or Kiswahili. The ACASI programme included built-in skip patterns, and logic checks. Upon completion of each ACASI interview, six acceptability questions were asked in ACASI only.

Risk assessment in FtF-interview was conducted after socio-demographic questions were obtained. Counsellors had been trained on asking risk questions and providing explanations to certain questions during the start of the cohort (July 2005), and were familiar with the questionnaire as it also was used for follow up visits.

### Data management and analysis

Questionnaire data was entered and stored in a secure database. Quality of data recorded was scrutinised by external data monitors, twice a month. Prior to data analysis, all site database entries were individually compared to source documents. Data cleaning, coding and analysis were conducted using Stata 9.2 [26].

The Chi-squared test and Student t-test were used to determine associations between categorical and continuous values, respectively. Differences in paired ACASI and FtF-responses were compared using the McNemar's chi squared test for binary data, and the Wilcoxon matched-pairs signed-rank test or paired Student t-test for continuous variables as appropriate [27]. Inter-method agreement was assessed by calculation of the Cohen's kappa statistic for binary and categorical variables, and Spearman's correlation coefficient for continuous variables. For categorical variables that had significant differences between interview methods the effect of interview order was assessed using simple test of proportions. For continuous responses Bland Altman plots were used to inspect for differences in variability between methods and the effect of the order of interview methods [28].
